# Genome Context Influences Evolutionary Flexibility of Nearly Identical Type III Effectors in Two Phytopathogenic Pseudomonads

**DOI:** 10.3389/fmicb.2022.826365

**Published:** 2022-02-18

**Authors:** David A. Baltrus, Qian Feng, Brian H. Kvitko

**Affiliations:** ^1^School of Plant Sciences, University of Arizona, Tucson, AZ, United States; ^2^School of Animal and Comparative Biomedical Sciences, University of Arizona, Tucson, AZ, United States; ^3^Institute of Plant Breeding, Genetics and Genomics, University of Georgia, Athens, GA, United States; ^4^Department of Plant Pathology, University of Georgia, Athens, GA, United States

**Keywords:** *Pseudomonas syringae*, integrative conjugative element (ICE), type III effector, phytopathogen, *Nicotiana benthamiana*

## Abstract

Integrative Conjugative Elements (ICEs) are replicons that can insert and excise from chromosomal locations in a site-specific manner, can conjugate across strains, and which often carry a variety of genes useful for bacterial growth and survival under specific conditions. Although ICEs have been identified and vetted within certain clades of the agricultural pathogen *Pseudomonas syringae*, the impact of ICE carriage and transfer across the entire *P. syringae* species complex remains underexplored. Here we identify and vet an ICE (PmaICE-DQ) from *P. syringae* pv. maculicola ES4326, a strain commonly used for laboratory virulence experiments, demonstrate that this element can excise and conjugate across strains, and highlight that this element contains loci encoding multiple type III effector proteins. Moreover, genome context suggests that another ICE (PmaICE-AOAB) is highly similar in comparison with and found immediately adjacent to PmaICE-DQ within the chromosome of strain ES4326, and also contains multiple type III effectors. Lastly, we present passage data from *in planta* experiments that suggests that genomic plasticity associated with ICEs may enable strains to more rapidly lose type III effectors that trigger R-gene mediated resistance in comparison to strains where nearly isogenic effectors are not present in active ICEs. Taken together, our study sheds light on a set of ICE elements from *P. syringae* pv. maculicola ES4326 and suggests how genomic context may lead to different evolutionary dynamics for shared virulence genes between strains.

## Introduction

Genome fluidity is crucial for survival of bacterial phytopathogens, as selection pressures on the presence and function of specific virulence genes can dramatically change from host to host (Dillon et al., 2019a; de Vries et al., 2020). Much research characterizing gene composition of bacterial pathogens focuses on presence and absence of specific virulence genes, and therefore often extrapolates from lists of loci to predict virulence and evolutionary potential (Baltrus et al., 2011; Dillon et al., 2019b). However, even if virulence genes are conserved across strains of a particular pathogen, differences in genomic flexibility for shared virulence genes could potentiate different evolutionary outcomes between strains under conditions of strong selection. Here we characterize one instance where such differences in genomic context and fluidity for a set of type III effectors affects evolutionary potential for *Pseudomonas syringae*, and we speculate about the ability of such systems to shift evolutionary dynamics for phytopathogens moving forward.

Integrative Conjugative Elements (ICEs) are mobile replicons that blend characteristics of both plasmids and prophage and are well known for their ability to harbor antibiotic resistance genes and other niche-association traits across bacteria (Johnson and Grossman, 2015). Like conjugative plasmids, ICEs contain all genes and pathways required for conjugation between bacterial cells. Like prophage, ICEs contain site specific recombinases that enable recombination into chromosomal locations and repress the genes responsible for conjugation and replication while in this quiescent state. Most importantly, ICEs also contain cargo regions that can house genes and pathways that directly contribute to dramatic changes in bacterial phenotypes (Lovell et al., 2009; Johnson and Grossman, 2015). Not only can these elements modify phenotypes in their current host cells, but the ability of ICEs to transfer throughout populations and across communities through horizontal gene transfer means that cargo genes present on ICEs can rapidly proliferate across strains if they are beneficial. The rise of sequencing technologies enabling closed bacterial genomes has reinforced the importance of mobile elements in genomes and increased awareness of the prevalence of ICEs throughout bacteria.

The phytopathogen *Pseudomonas syringae* (sensu lato) is a recognized agricultural pest for many crops throughout the world, with numerous strains well established as laboratory systems for understanding virulence of plant pathogens *in vitro* and *in planta* (Baltrus et al., 2017). The presence of a type III secretion system is critical for virulence *in planta* for many strains of this pathogen, and this system is used to translocate upwards of 40 effectors proteins per strain from the bacterial cytoplasm into plant cells (Dillon et al., 2019a; Laflamme et al., 2020). Once inside the plant cells, effector proteins can disrupt plant immune responses in a variety of ways to promote bacterial growth and infection. The presence of effector proteins can also be monitored by plant immune responses through the action of R-genes, with recognition of effector protein functions leading to an overarching immune reaction termed effector triggered immunity (ETI) (Collmer et al., 2000; Jones and Dangl, 2006; Jayaraman et al., 2021). Triggering of the ETI response can quickly shut down nascent infections in resistant plant cultivars and has thus formed the basis of future plans to engineer durable crop resistance to infection through genetic modification and selective breeding (Dong and Ronald, 2019; Laflamme et al., 2020). Thus, type III effectors sit at an evolutionary inflection point where they can be highly beneficial for bacterial growth in some host backgrounds and highly detrimental in others.

Throughout this manuscript, we use the phrase “genome context” as a catch-all term to represent both the placement and relative location of specific genes within a genome compared to orthologs and homologs of these specific genes. We intend this as an open-ended term to describe how sequence characteristics (gene order, location, etc.) can affect parameters that contribute to evolutionary potential. For instance, imagine two antibiotic resistance genes that are sequence identical and found within a single genome but where one copy is present on a chromosome and stably maintained whereas the other copy is found in a region that is much more easily lost (e.g., on a small mobilizable plasmid). While these two genes may play similar if not redundant phenotypic roles for cells containing this particular version of the genome, differences between copies in rates of loss and horizontal transfer could significantly influence the evolutionary potential of each copy across the population and microbial community by altering population genetic parameters like population size and deletion rates.

The presence of virulence genes, including type III effectors, on plasmids and consequent movement across strains can dramatically alter trajectories of virulence evolution for *P. syringae* on different host species (Cazorla et al., 2002; Schierstaedt et al., 2019). Plasmids can be lost or modified if effector proteins are recognized by a potential host, which could enable infections to proceed at a population level despite the ETI response (Grant et al., 2006; Bardaji et al., 2019). Plasmids are not the only element within *P. syringae* genomes displaying plasticity, though. For instance, the effector AvrPphB (aka HopAR1) triggers HR in bean plants that contain the R-gene RPS5 (Qi et al., 2014). AvrPphB is found on an genomic island in certain strains of *P. syringae* pathovar phaseolicola, and under selective pressure from ETI responses, bacteria with the island excised from the chromosome and maintained independently as an episome rapidly dominate the population (Godfrey et al., 2011; Neale et al., 2018). Excision of the genomic island containing AvrPphB prevents ETI because this Avr gene is downregulated under episomal replication, but enables this gene to be maintained within a population for infection of host plants where it may be beneficial. ICE elements have also been identified and characterized in *P. syringae* pathovar actinidae strains where their presence/absence is one of the most glaring differences between closely related strains and where they contribute to large scale differences in strain metabolism and resistance to antibacterial compounds (Colombi et al., 2017; McCann et al., 2017; Poulter et al., 2018).

Here we describe how genomic context in a locus encoding an effector protein, HopQ, differentially affects evolutionary flexibility for strains containing this effector. Recognition of HopQ by the plant R-gene Roq1 triggers ETI in the host plant *Nicotiana benthamiana*, and thus limits growth of strain *P. syringae* pv. tomato DC3000 (*Pto)* in this host (Wei et al., 2007; Schultink et al., 2017). We characterize the genomic context for *hopQ* and other linked virulence genes in both *Pto*DC3000 and another pathogen, *Pseudomonas syringae* pv. maculicola ES4326 (*Pma*ES4326). We demonstrate that this and other effectors are differentially present in an active ICE element in *Pma*ES4326 but not in *Pto*DC3000, and confirm that recognition of HopQ also limits growth of *Pma*ES4326 in *N. benthamiana*. These differences are somewhat surprising because at a broad scale these regions appear syntenic. We further show how this differential genomic context allows for differential genomic plasticity for *hopQ* between these two strains, by demonstrating that passage of strains in *Nicotiana benthamiana* leads *hopQ* to be more readily lost in *Pma*ES4326 than in *Pto*DC3000. Thus, we directly demonstrate how genomic context for homologous virulence genes in two closely related pathogens can contribute to different evolutionary trajectories for these strains.

## Materials And Methods

### Bacterial Strains and Culturing Conditions

*E. coli* was routinely cultured at 37°C in LB (Lysogeny broth; per 1 L = 10 g tryptone, 5 g yeast extract, 10 g NaCl, pH 7.5 + with 15 g agar for solidified media). *P. syringae* was routinely cultured at 25°C or 28°C in either LM (LB modified; per 1 L = 10.0 g tryptone, 6.0 g yeast extract, 2.0 g K_2_HPO_4_⋅3H_2_O, 0.6 g NaCl, 0.4 g MgSO_4_⋅7H_2_O, with 18 g agar for solidified media), KB (King’s B; per 1 L = 20.0 g proteose peptone 3, 0.4 g MgSO_4_⋅7H_2_O, glycerol 10 mL, 2.0 g K_2_HPO_4_⋅3H_2_O, with 18 g agar for solidified media) or KBC (KB amended with boric acid 1.5 g/L and cephalexin 80 mg/L). Liquid cultures were incubated with shaking at 200 rpm. Where appropriate, media was augmented at final concentration with rifampicin (Rf) 40–60 μg/mL, gentamicin (Gm) 10 μg/mL, kanamycin (Km) 50 μg/mL, spectinomycin (Sp) 50 μg/mL, and diaminopimelic acid (DAP) 200 μg/mL for liquid media, 400 μg/mL for solid media for the growth of DAP-auxotrophic *E. coli* strains.

### Genome Sequencing and Assembly

For each strain with a genome sequence reported herein, a frozen stock was streaked to single colonies on King’s B (KB) agar plates, at which point a single colony was picked to 2 mL KB liquid and grown overnight on a shaker at 27°C. Genomic DNA was extracted from these overnight cultures using a Promega (Madison, WI) Wizard kit and including the optional RNAse step. Each strain was sequenced using multiple technologies, and in each case independent DNA isolations were used to prepare libraries for different sequencing platforms. Illumina sequencing for strains was performed by MiGS (Pittsburgh, PA) using their standard workflow. As described in Baym et al. (2015), this workflow uses an Illumina tagmentation kit for library generation, followed by sequencing on a NextSeq 550 instrument with 150-bp paired-end reads. Trimmomatic was used for adaptor trimming (Bolger et al., 2014). For nanopore sequencing for strain *Pma*ES4326-D, a library was prepared from unsheared DNA using the Rapid sequencing kit (SQK-RAD004) and sequenced on a Flongle flowcell. For nanopore sequencing for strains *Pma*ES4326-C, *Pma*ES4326ΔDQ3, and *Pma*ES4326-C-LA-P5-20-1, each library was prepared using unsheared DNA as an input to the LSK109 ligation sequencing kit and was sequenced on R9.4 MinION flowcells. Nucleotide bases were called during sequencing using Guppy v3.2.6 in Fast-Mode. All genomes were assembled using Unicycler v.0.4.8 (Wick et al., 2017). The public facing genomes for *Pma*ES4326-D and *Pma*ES4326-C were annotated using PGAP (Tatusova et al., 2016). Genomes for *Pma*ES4326ΔDQ3, and *Pma*ES4326-C-LA-P5-20-1 found in Figshare doi: 10.6084/m9.figshare.17064080 were annotated using Prokka v 1.14.6 (Seemann, 2014). We used breseq v. 0.35.7 (Deatherage and Barrick, 2014) to identify evolutionary changes that occurred in the passage strain *Pma*ES4326 LA-P5-20-1 (hereafter *Pma*ES4326 LAP5-20). Default parameters were used for all software unless otherwise specified.

### Integrative Conjugative Element Identification

Potential ICE elements were identified by manual searches of gene annotations for loci that could code for proteins critical for processing of conjugative elements. These include a site specific recombinase, a TraG-like NTPase, an ATPase, and numerous proteins involved in creation of the conjugation pilus. To be present within an ICE, these elements must all be present in a contiguous segment of the genome which is bordered by a potential attachment site (often tRNA loci).

### DNA Manipulation

Plasmid DNA was routinely purified using the GeneJet plasmid miniprep kit (Thermo Fisher Scientific). PCR was conducted with Phusion HiFi polymerase (Thermo Fisher Scientific). PCR/reaction cleanup and gel extraction were conducted with the Monarch PCR and DNA cleanup kit and Monarch DNA gel extraction kits (NEB). *E. coli* transformation was conducted by either by preparing competent cells using the Mix and Go! *E. coli* transformation kit (Zymo Research) or standard electro-transformation protocols. *Pma*ES4326 strains were transformed with pCPP5372hopQ (pHopQ) (Wei et al., 2018) plasmid DNA via electro-transformation after washing and concentration of overnight liquid cultures with 300 mM sucrose (Choi et al., 2006). Restriction enzymes, T4 ligase, and Gibson Assembly Mastermix were purchased from NEB. Oligonucleotide primers were synthesized by IDT. Commercial molecular biology reagents were used in accordance with their manufacturer’s recommendations.

To create the site-specific Tn*7* 3xmCherry labeling vector pURR25DK-3xmcherry, pURR25 (Teal et al., 2006) was first digested with *Pst*I and recircularized with T4 ligase to remove the *nptII* Km^R^ marker gene creating pURR25DK. To replace the *gfp* gene in pURR25DK, the 3xmCherry cassette was PCR amplified from pGGC026 (pGGC026 was a gift from Jan Lohmann (Addgene plasmid # 48831^1^; RRID:Addgene_48831) using primers bko374 (5′**ACATCTAGAATTAAAGAGGAGAAATTAAGCATGGTG AGCAAGGGCGAGGAGGATAACATG 3**′**) and** bko375 (5′**CAGGAGTCCAAGCTCAGCTAATTAAGCTTACTTGTAC AACTCATCCATACCACCTGTTGA 3**′) to introduce 30 bp 5′ overlaps corresponding to the *gfp* flanking regions. Gibson assembly was used to join the 3xmCherry PCR amplicon with pURR25DK backbone digested with *Bse*RI and partially digested with *Hin*dIII.

#### Bacterial Conjugation and Creation of Mutant Strains

All genetic manipulations of *Pma*ES4326 were conducted with the *Pma*ES4326-C strain. Conjugations were performed by mixing 15 μL of fivefold concentrated, washed, overnight LM liquid cultures of each parent strain and co-culturing at 28°C overnight on sterile nitrocellulose membranes on either LM or LM + DAP plates (for conjugations with DAP-auxotrophic *E. coli* donor strains). Tn*7* transposition conjugations always included the *E. coli* RHO3 pTNS3 Tn*7* transposase helper strain as a third parent. For all conjugation experiments cultures of each parent strain were included separately as controls. *Pma*ES4326 merodiploid exconjugants of pCPP5729 (pK18msGmΔ*hopQ1)* were recovered on LM Km (Kvitko et al., 2009). Resolved Pma ES4326 pCPP5729 merodiploids were recovered via counter-selection on LM Rf + 10% sucrose and sucrose resistant clones were screened for kanamycin sensitivity by patch plating indicating the loss of the pK18ms plasmid backbone (Kvitko and Collmer, 2011). Derivative *att*Tn*7*-3xmCherry transposant strains were recovered on LM Sp and pink clones were selected after 4 days incubation at 4°C and restreaked to isolation. To test the native mobility of ICE-DQ, conjugation was conducted as described above with the *Pma*ES4326 pCPP5729 merodiploid as the donor parent and *att*Tn*7*-3xmCherry derivatives of *Pto*DC3000 and *Pma*ES4326 ΔICE-DQ strains (*Pma* DQ3 and *Pma* LAP5-20) as recipients. ICE-DQ exconjugants were recovered on LM Rf Sp Km. For all conjugation experiments cultures of each parent strain were included separately as controls. Conjugation frequency was calculated as the number of Sp^R^Km^R^ colonies recovered per recipient CFU as determined by dilution plating.

#### *Nicotiana benthamiana* Growth, Inoculation and Bacterial Passage Assays

*Nicotiana benthamiana*, WT LAB accession, and *roq1-1* (Qi et al., 2018) were grown in a Conviron Adaptis growth chamber with 12 h light (125 μmol/m^2^/s) at 26°C and 12 h dark at 23°C. Plants were used at 5-7 weeks post germination. To prepare inoculum, *P. syringae* cultures were recovered from fresh KB plate cultures, resuspended in 0.25 mM MgCl_2_, standardized to OD_600_ 0.2, and serially diluted 10,000X to ∼3 × 10^4^ CFU/mL. Cell suspensions were infiltrated with a blunt syringe into either the 3rd, 4^th^, or 5th leaves. Infiltrated spots were allowed to dry fully and then plants were covered with a humidity dome and kept at 100% humidity for 6–8 days to allow symptoms to develop. At end point, leaves were photographed to document symptoms and four 4 mm diameter leaf punches (∼0.5 cm^2^ total) were collected with a 4 mm diameter biopsy punch from each infiltrated area. Discs were macerated in 0.1 mL of 0.25 mM MgCl_2_ using an Analytik Jena SpeedMill Plus homogenizer and the bacterial CFU/cm^2^ leaf tissue was determined by serial dilution spot plating from 10 μL volumes on LM Rf.

For *P. syringae* passaging in *N. benthamiana*, single colonies of *Pto* DC3000 WT and *Pma*ES4326 were inoculated into LM Rf liquid cultures. Samples of the initial cultures (P0) were cryo-preserved at −80°C in 15% final strength sterile glycerol. The liquid cultures were diluted 1000X in 0.25 mM MgCl_2_ to approximately 5 × 10^5^ CFU/mL prior to syringe infiltration into three leaf areas establishing three lineages (A, B, C) each for *Pto*DC3000 and *Pma*ES4326. Tissue samples were collected 6–7 days post inoculation and processed as described above. Bacteria cultured from the 10^–1^ tissue macerate dilutions of each lineage were directly scraped from the dilution plate with an inoculation loop and suspended in 1 mL 0.25 mM MgCl_2_. These suspensions were then sub-cultured 5 μL into 5 mL LM Rf and diluted as described above to create inoculum for the next passage. This passaging scheme was repeated five times and samples of both the post-passage recovered bacteria (P1) and the corresponding sub-cultured bacteria (P1c) were cryo-preserved for each lineage and each passage. To screen for changes in *N. benthamiana* disease compatibility with passaged strains in a medium-throughput format, bacteria from the P5 cryo-preserved samples were streaked to isolation on KBC plates and isolated “P5” colonies were cultured in 200 μL of LM Rf in sterile 96 well microtiter plates along with Pto DC3000, *Pto* DC3000Δ*hopQ*, *Pma* ES4326 and *Pma*ES4326ΔDQ3 control strains. Cultures were serially diluted 5000X in 0.25 mM MgCl_2_ to ∼3 × 10^4^ CFU/mL and inoculated into *N. benthamiana* leaves as described. Inoculum concentration was verified via serial dilution spot plating. Inoculated areas were monitored visually for qualitative changes to disease symptoms compared to their respective WT and targeted *hopQ* deletion control strains over the course to 6–8 days. The CFU/cm^2^ leaf tissue bacterial load of select clones was determined for each strain as described above. Isolated colonies of strains that displayed bacterial load and symptoms comparable to their respective Δ*hopQ* backgrounds were subcultured from the dilution spot count plates and retained.

We estimate that N_e_ of these passage populations to be on the order of 2.5 × 10^4^ over the course of five passages. This number was calculated by taking the inoculum of each passage (5 × 10^5^ CFU/mL in 50 μL) and estimating that approximately 10 μL were inoculated per cm^2^ for a total of 2.5 × 10^4^ for the area inoculated (∼5 cm^2^). We further estimate that this is the smallest bottleneck over the course of the passages, and that populations undergo ∼10 generations of division, as these populations grow from 10^4^ to 10^7^ cm^2^ *in planta*). Given these assumptions, N_e_ can be estimated by taking the harmonic mean of fluctuating population sizes (Sjödin et al., 2005) and will be highly skewed by smaller numbers. Since 1 cm^2^ is harvested at the end of each cycle, a conservative estimate for N_e_ over five passages is therefore approximately 2.5 × 10^4^ CFU.

## Results

### Complete Genome Sequences for Multiple Isolates of *Pma*ES4326

We previously reported a draft genome sequence for an isolate of *Pma*ES4326 acquired from the lab of Jeff Dangl (Baltrus et al., 2011), and our first goal was to generate a complete genome sequence for this strain (referred to herein as *Pma*ES4326-D, Table 1). We used MiGS (Pittsburgh, PA) to generate Illumina reads for *Pma*ES4326-D, and their workflow generated a total of 3,284,990 paired reads and 418 Mb (∼64× coverage) of sequence. We also independently isolated genomic DNA and sequenced using an Oxford Nanopore MinION to generate 32,291 reads for a total of 465 Mb (∼71× coverage) of sequence with a read N50 of 30,656 bp. Assembly of these reads resulted in a complete circular chromosome and four separate plasmids. Notably, our isolate of *Pma*ES4326-D contains three previously reported plasmids (pPma4326A, pPma4326B, pPma4326E) but also appears to have lost two different plasmids (pPma4326C and pPma4326D) first reported as present in this strain by Stavrinides and Guttman (2004). The assembly for this strain appears to contain an additional ∼350 kb plasmid that was not reported by Stavrinides and Guttman (2004) and which we name pPma4326F following earlier naming conventions.

**TABLE 1:** Sequencing of *Pma*ES4326 strains.

Strain	Reads (Illumina/Nanopore)	Total bp sequenced Mb (Illumina/Nanopore)	Read N50 Nanopore bp	Total sequencing depth (Illumina/Nanopore)	SRA accession (Illumina/Nanopore)
*Pma*ES4326-D	3,284,990/32,291	418.372/465.212	30,656	64×/71×	SRR1598872 SRR15988724
*Pma*ES4326-C	3,757,284/55,845	485.469/700.483	23,669	74×/107×	SRR15988571/ SRR15988568
*Pma*ES4326ΔDQ3	2,479,212/15,275	331.276/178.118	31,491	51×/27×	SRR15988570/ SRR15988567
*Pma*ES4326-C-LA-P5-20-1	1,372,720/9,934	189.971/146.897	31,020	29×/22×	SRR17005742/ SRR15988569

Given the absence of two plasmids from *Pma*ES4326-D, we sought to generate a genome assembly from a different lab isolate, acquired from the lab of Alan Collmer referred to here as *Pma*ES4326-C (Table 1). We used MiGS (Pittsburgh, PA) to generate Illumina reads for the Collmer lab version of *Pma*ES4326, and their workflow generated a total of 3,757,284 paired reads and 485 Mb (∼74× coverage) of sequence. We also independently isolated genomic DNA and sequenced using an Oxford Nanopore MinION to generate 55,845 reads for a total of 700 Mb (∼107× coverage) of sequence with a read N50 of 24,669 bp. Hybrid assembly of all read types resulted in a single chromosome that appears nearly complete but was not circularized by Unicycler. However, this assembly does contain all predicted circular plasmids as well as pPmaES4326F.

Sequencing and assembly characteristics for all strains can be found in Tables 1, 2, respectfully.

**TABLE 2:** Assembly of *Pma*ES4326 strains.

Strain	Complete genome	Number of contigs in assembly	Replicons present	Assembly accession
*Pma*ES4326-D	Yes	5	Chromosome (circular), pPmaES4326ABEF	GCA_000145845.2
*Pma*ES4326-C	Yes	7	Chromosome (circular), pPmaES4326ABCDEF	GCA_020309905.1
*Pma*ES4326ΔDQ3	Yes	6	Chromosome (circular), pPmaES4326ABCF	doi: 10.6084/m9.figshare.17064080
*Pma*ES4326-C-LA-P5-20-1	No	7	Chromosome (not circular), pPmaES4326ABCDEF	doi: 10.6084/m9.figshare.17064080

### An Integrative Conjugative Element Hotspot in *Pma*ES4326

**FIGURE 1:**
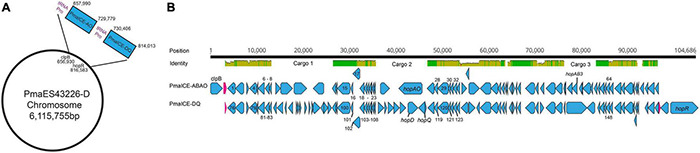
Comparison of PmaICE-ABAO and PmaICE-DQ. **(A)** A representation of the circular chromosome of strain *Pma*ES4326-D, highlighting the region where both ICE elements are found as well as the tRNA-Pro loci that border these ICE elements. **(B)** We aligned nucleotide sequences from the pair of potential ICEs found within the *clpB-hopR* region in strain *Pma*ES4326 against each other, and visualize the results here. The top line of the figure displays overall nucleotide identity between these two ICEs, with green bars representing conserved nucleotides and yellow representing slight to modest divergence. If no colored bars on this top line, the sequences are completely divergent, and we highlight the three potential cargo regions that differ between these ICE elements. Predicted loci for each ICE are shown on the lines immediately below the nucleotide identity comparison. *Pma*ICE-ABAQ is positioned immediately adjacent to *clpB* in the *Pma*ES4326 genome while *Pma*ICE-DQ is positioned immediately adjacent to *hopR* in the same genome. Each predicted locus is represented in the figure in blue, while predicted tRNA loci are represented in magenta. We labeled all identified type III effector loci within these regions as well as *clpB*. All loci that are implicated in ICE function, by annotation, have been labeled with numbers that correspond to their annotations within Table 3.

Genomic inspection of the region containing type III effectors *hopQ* and *hopD* in strains *Pma*ES4326-C and *Pma*ES4326-D indicate that this area is a potential hotspot for genomic plasticity. Specifically, in a region bordered by *clpB* and the type III effector *hopR*, gene content characterization strongly suggests the presence of two independent integrative conjugative elements (ICEs, Figure 1). Both ICEs are approximately 70 kb in length, and are found adjacent to tRNA loci encoding a proline anticodon. Moreover, roughly 70% of each element is composed of sequences with > 95% nucleotide similarity and which encode many of the structural genes predicted to be involved in ICE proliferation and integration. These conserved genes include predicted integrases/recombinases, pilus proteins and ATPases, regulator proteins, a topoisomerase (*topB*) and helicase, chromosome partitioning proteins (*parB*), DNA coupling proteins (*traD*), an NTPase (*traG*), and numerous loci annotated as “integrative conjugative element proteins.” We have included annotations for all genes within these two ICEs in Table 3.

**TABLE 3:** Gene annotations in *Pma*ES4326 ICEs.

Number	Name	Type	Minimum	Maximum	Length	Direction
	tRNA-Asn	tRNA	2,862	2,937	76	Forward
	tRNA-Pro	tRNA	2,967	3,043	77	Forward
	tRNA-Lys	tRNA	3,049	3,124	76	Forward
	tRNA-Pro	tRNA	3,220	3,296	77	Forward
1	Tyrosine-type recombinase/integrase	CDS	3,625	5,064	1,440	Reverse
2	DNA-binding domain-containing protein	CDS	5,061	6,992	1,932	Reverse
3	DUF3742 family protein	CDS	7,604	8,005	402	Forward
4	*traG* conjugal transfer protein	CDS	8,047	9,585	1,539	Reverse
5	Hypothetical protein	CDS	9,582	9,950	369	Reverse
6	Integrating conjugative element protein	CDS	9,953	11,323	1,371	Reverse
7	TIGR03756 family integrating conjugative elementprotein	CDS	11,344	12,282	939	Reverse
8	TIGR03757 family integrating conjugative elementprotein	CDS	12,279	12,755	477	Reverse
9	Hypothetical protein	CDS	13,053	13,241	189	Forward
10	Hypothetical protein	CDS	13,259	13,798	540	Forward
11	Hypothetical protein	CDS	13,839	14,393	555	Forward
12	Hypothetical protein	CDS	14,752	15,297	546	Reverse
13	Thioredoxin domain-containing protein	CDS	15,609	16,307	699	Reverse
14	Hypothetical protein	CDS	16,304	16,603	300	Reverse
15	Conjugative transfer ATPase	CDS	16,600	19,347	2,748	Reverse
16	TIGR03751 family conjugal transfer lipoprotein	CDS	19,557	20,012	456	Reverse
17	TIGR03752 family integrating conjugative elementprotein	CDS	19,990	21,486	1,497	Reverse
18	TIGR03749 family integrating conjugative elementprotein	CDS	21,476	22,399	924	Reverse
19	TIGR03746 family integrating conjugative elementprotein	CDS	22,396	23,064	669	Reverse
20	TIGR03750 family conjugal transfer protein	CDS	23,061	23,453	393	Reverse
21	TIGR03745 family integrating conjugative elementmembrane protein	CDS	23,469	23,837	369	Reverse
22	TIGR03758 family integrating conjugative elementprotein	CDS	23,857	24,096	240	Reverse
23	Conjugal transfer protein	CDS	24,093	24,458	366	Reverse
24	DUF4177 domain-containing protein	CDS	24,538	24,870	333	Reverse
25	Hypothetical protein	CDS	25,041	25,472	432	Forward
26	*hopAO* type III effector	CDS	25,861	26,877	1,017	Reverse
27	UvrD-helicase domain-containing protein	CDS	27,062	28,519	1,458	Reverse
28	TIGR03747 family integrating conjugative elementmembrane protein	CDS	28,529	29,278	750	Reverse
29	*traD*	CDS	29,318	31,477	2,160	Reverse
30	Integrating conjugative element protein	CDS	31,489	31,992	504	Reverse
31	Transglycosylase SLT domain-containing protein	CDS	31,989	32,546	558	Reverse
32	TIGR03759 family integrating conjugative element protein	CDS	32,531	33,277	747	Reverse
33	Hypothetical protein	CDS	33,286	33,990	705	Reverse
34	*dcm*	CDS	34,335	35,390	1,056	Forward
35	*vsr*	CDS	35,329	35,766	438	Reverse
36	DNA mismatch repair protein	CDS	35,850	37,871	2,022	Forward
37	DEAD/DEAH box helicase family protein	CDS	37,973	40,231	2,259	Reverse
38	Class I SAM-dependent methyltransferase	CDS	40,335	41,786	1,452	Reverse
39	Hypothetical protein	CDS	41,816	42,415	600	Reverse
40	DUF3275 family protein	CDS	42,536	43,147	612	Reverse
41	Hypothetical protein	CDS	43,248	43,610	363	Reverse
42	Hypothetical protein	CDS	43,672	43,986	315	Reverse
43	DUF3577 domain-containing protein	CDS	44,290	44,859	570	Reverse
44	Hypothetical protein	CDS	44,879	45,403	525	Forward
45	Hypothetical protein	CDS	45,480	45,848	369	Reverse
46	Regulator	CDS	46,056	46,589	534	Reverse
47	*topB*	CDS	47,650	49,671	2,022	Reverse
48	Hypothetical protein	CDS	49,794	50,363	570	Reverse
49	Hypothetical protein	CDS	50,657	51,094	438	Reverse
50	ATP-dependent helicase	CDS	51,329	53,281	1,953	Reverse
51	Hypothetical protein	CDS	53,284	54,849	1,566	Reverse
52	CrpP family protein	CDS	55,287	55,466	180	Reverse
53	Hypothetical protein	CDS	55,471	55,644	174	Reverse
54	AAA family ATPase	CDS	55,991	56,941	951	Reverse
55	*hopAB* type III effector	CDS	56,958	58,115	1,158	Reverse
56	IS91 family transposase	CDS	58,481	59,101	621	Reverse
57	RulB protein	CDS	59,132	59,200	69	Reverse
58	IS481 family transposase	CDS	59,217	60,878	1,662	Reverse
59	Recombinase family protein	CDS	60,859	61,434	576	Reverse
60	Hypothetical protein	CDS	61,751	62,224	474	Reverse
61	Single-stranded DNA-binding protein	CDS	62,425	62,886	462	Reverse
62	Phage regulatory protein	CDS	62,898	63,730	833	Reverse
63	DUF3158 family protein	CDS	63,736	64,257	522	Reverse
64	TIGR03761 family integrating conjugative elementprotein	CDS	64,294	65,073	780	Reverse
65	Hypothetical protein	CDS	65,066	65,662	597	Reverse
66	Hypothetical protein	CDS	65,992	67,353	1,362	Reverse
67	DUF2857 family protein	CDS	67,350	68,087	738	Reverse
68	*parB*chromosome partitioning protein	CDS	68,124	69,884	1,761	Reverse
69	Arc family DNA-binding protein	CDS	69,887	70,204	318	Reverse
70	Hypothetical protein	CDS	70,382	70,786	405	Reverse
71	Hypothetical protein	CDS	70,788	71,117	330	Reverse
72	Hypothetical protein	CDS	71,437	72,123	687	Reverse
73	Hypothetical protein	CDS	72,563	73,153	591	Reverse
74	*dnaB*	CDS	73,150	74,496	1,347	Reverse
75	AAA family ATPase	CDS	74,554	75,414	861	Reverse
	tRNA-Pro	tRNA	75,651	75,727	77	Forward
76	Tyrosine-type recombinase/integrase	CDS	76,041	77,495	1,455	Reverse
77	DNA-binding domain-containing protein	CDS	77,492	79,423	1,932	Reverse
78	DUF3742 family protein	CDS	80,037	80,438	402	Forward
79	*traG* conjugal transfer protein	CDS	80,499	82,031	1,533	Reverse
80	Hypothetical protein	CDS	82,028	82,402	375	Reverse
81	Integrating conjugative element protein	CDS	82,405	83,772	1,368	Reverse
82	TIGR03756 family integrating conjugative elementprotein	CDS	83,793	84,731	939	Reverse
83	TIGR03757 family integrating conjugative elementprotein	CDS	84,728	85,159	432	Reverse
84	DUF1440 domain-containing protein	CDS	85,713	86,201	489	Reverse
85	Lytic murein transglycosylase	CDS	86,453	87,694	1,242	Reverse
86	Hypothetical protein	CDS	88,186	88,395	210	Reverse
87	IS5 family transposase	CDS	88,841	89,818	978	Reverse
88	Carbon storage regulator	CDS	90,586	90,810	225	Forward
89	TraR/DksA family transcriptional regulator	CDS	90,846	91,262	417	Forward
90	Hypothetical protein	CDS	91,297	91,518	222	Forward
91	GNAT family N-acetyltransferase	CDS	92,339	92,830	492	Forward
92	AAA family ATPase	CDS	93,208	93,489	282	Forward
93	*tnpB*	CDS	93,794	94,150	357	Forward
94	IS66 family transposase	CDS	94,167	95,699	1,533	Forward
95	IS5 family transposase	CDS	95,741	96,382	642	Reverse
96	AAA family ATPase	CDS	96,453	97,271	819	Forward
97	(p)ppGpp synthetase	CDS	97,274	98,092	819	Forward
98	Thioredoxin domain-containing protein	CDS	98,266	98,964	699	Reverse
99	Hypothetical protein	CDS	98,961	99,260	300	Reverse
100	Conjugative transfer ATPase	CDS	99,257	102,004	2,748	Reverse
101	TIGR03751 family conjugal transfer lipoprotein	CDS	102,214	102,669	456	Reverse
102	TIGR03752 family integrating conjugative elementprotein	CDS	102,647	104,143	1,497	Reverse
103	TIGR03749 family integrating conjugative elementprotein	CDS	104,133	105,056	924	Reverse
104	TIGR03746 family integrating conjugative elementprotein	CDS	105,053	105,721	669	Reverse
105	TIGR03750 family conjugal transfer protein	CDS	105,718	106,110	393	Reverse
106	TIGR03745 family integrating conjugative elementmembrane protein	CDS	106,126	106,494	369	Reverse
107	TIGR03758 family integrating conjugative elementprotein	CDS	106,514	106,753	240	Reverse
108	Conjugal transfer protein	CDS	106,750	107,115	366	Reverse
109	DUF4177 domain-containing protein	CDS	107,195	107,525	331	Reverse
110	MFS transporter	CDS	108,102	109,286	1,185	Forward
111	Transposase	CDS	109,422	109,937	516	Forward
112	Amidinotransferase	CDS	110,235	111,335	1,101	Forward
113	Serine kinase - nikkomycin	CDS	111,422	112,675	1,254	Forward
114	Serine kinase	CDS	112,728	113,972	1,245	Forward
115	*hopD*type III effector protein	CDS	114,438	116,555	2,118	Forward
116	*hopQ*type III effector protein	CDS	116,671	118,014	1,344	Reverse
117	Phosphoribulokinase	CDS	118,048	118,365	318	Forward
118	UvrD-helicase domain-containing protein	CDS	118,603	120,060	1,458	Reverse
119	TIGR03747 family integrating conjugative elementmembrane protein	CDS	120,070	120,819	750	Reverse
120	*traD*	CDS	120,859	123,018	2,160	Reverse
121	Integrating conjugative element protein	CDS	123,030	123,536	507	Reverse
122	Tansglycosylase SLT domain-containing protein	CDS	123,533	124,090	558	Reverse
123	TIGR03759 family integrating conjugative elementprotein	CDS	124,075	124,821	747	Reverse
124	Hypothetical protein	CDS	124,830	125,534	705	Reverse
125	IS5 family transposase	CDS	125,711	126,561	851	Reverse
126	Hypothetical protein	CDS	126,700	127,581	882	Forward
127	Hypothetical protein	CDS	127,745	128,107	363	Reverse
128	DEAD/DEAH box helicase family protein	CDS	128,202	130,460	2,259	Reverse
129	Class I SAM-dependent methyltransferase	CDS	130,564	132,015	1,452	Reverse
130	Hypothetical protein	CDS	132,045	132,644	600	Reverse
131	DUF3275 family protein	CDS	132,770	133,381	612	Reverse
132	Hypothetical protein	CDS	133,482	133,844	363	Reverse
133	DUF3577 domain-containing protein	CDS	134,172	134,726	555	Reverse
134	Hypothetical protein	CDS	134,746	135,270	525	Forward
135	Hypothetical protein	CDS	135,347	135,715	369	Reverse
136	Regulator	CDS	135,923	136,456	534	Reverse
137	*topB*	CDS	137,517	139,538	2,022	Reverse
138	Hypothetical protein	CDS	139,661	140,230	570	Reverse
139	Hypothetical protein	CDS	140,774	141,457	684	Forward
140	ATP-dependent helicase	CDS	141,779	143,731	1,953	Reverse
141	Hypothetical protein	CDS	143,734	145,259	1,526	Reverse
142	CrpP family protein	CDS	145,697	145,897	201	Reverse
143	IS3 family transposase	CDS	146,020	147,260	1,241	Forward
144	Hypothetical protein	CDS	147,274	147,519	246	Reverse
145	Single-stranded DNA-binding protein	CDS	147,725	148,186	462	Reverse
146	Phage regulatory protein	CDS	148,198	149,031	834	Reverse
147	DUF3158 family protein	CDS	149,037	149,600	564	Reverse
148	TIGR03761 family integrating conjugative elementprotein	CDS	149,597	150,376	780	Reverse
149	Hypothetical protein	CDS	150,369	150,918	550	Reverse
150	Hypothetical protein	CDS	151,362	152,723	1,362	Reverse
151	DUF2857 family protein	CDS	152,720	153,457	738	Reverse
152	*parB*chromosome partitioning protein	CDS	153,494	155,254	1,761	Reverse
153	Arc family DNA-binding protein	CDS	155,257	155,574	318	Reverse
154	Hypothetical protein	CDS	155,510	155,755	246	Reverse
155	Hypothetical protein	CDS	155,752	156,156	405	Reverse
156	Hypothetical protein	CDS	156,158	156,487	330	Reverse
157	Hypothetical protein	CDS	156,797	157,387	591	Reverse
158	*dnaB*	CDS	15,7384	158,730	1,347	Reverse
159	AAA family ATPase	CDS	158,788	159,648	861	Reverse
	tRNA-Pro	tRNA	159,887	159,963	77	Forward

Despite relatively high sequence similarity across these two closely related ICEs positioned successively in the genome of *Pma*ES4326, gene comparisons suggest the presence of three distinct cargo regions that clearly differentiate these elements (Figure 1). The first ICE contains type III effector proteins *hopAO* and *hopAB3-1* (aka *hopPmaL*) in two different predicted cargo regions, and so we name this region ICE-ABAO. The second ICE contains loci for type III effectors *hopD* and *hopQ* as well as the lytic transglycosylase *hopAJ1*, and so we name this ICE ICE-DQ. Cargo region one contains four predicted ORFs in *Pm*aECE-ABAO and fourteen predicted ORFs in ICE-DQ. Cargo region two contains two predicted ORFs in ICE-ABAO and seven predicted ORFs in ICE-DQ, including the effectors *hopAO*, *hopD*, and *hopQ.* Cargo region three contains six predicted ORFs in ICE-ABAO and two in ICE-DQ. Aside from the type III effectors, many of loci potentially encoded by these cargo regions are annotated as hypothetical proteins or as parts of IS elements and transposons.

### Differential Contexts for the Genomic Island Containing *hopD* and *hopQ* Across *Pma*ES4326 and *Pto*DC3000

**FIGURE 2:**
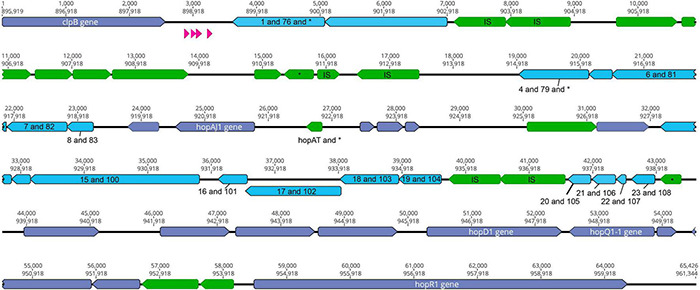
The *clpB-hopR* Region in *Pto*DC3000. Here we visualize the region between *clpB* and *hopR* from the *Pto*DC3000 genome, and compare this region to both predicted ICE elements from *Pma*ES4326. Each predicted locus is colored as purple (present in one of the *Pma*ES4326 ICE elements), blue (present in both *Pma*ES4326 ICE elements as per nucleotide identity), and green (only present in *Pto*DC3000). Predicted tRNA loci are shown in magenta. We also show two nucleotide positions within this region: top number is from the start of *clpB* and the bottom number is from the start of the genome (from *dnaA*). We label all identified type III effector loci and *clpB*, as well as all annotated IS elements (with an “IS”) and genes that have predicted frameshifts (with an “*”). All genes potentially implicated in ICE function have been labeled with numbers corresponding to potential orthologs from strain *Pma*ES4326 ICEs and listed in Table 3.

The type III effectors HopD and HopQ are nearly sequence identical in two phytopathogens commonly used as laboratory models for studying *P. syringae* virulence, *Pto*DC3000 and *Pma*ES4326 (Baltrus et al., 2011). Furthermore, these effectors are found in roughly the same genomic locations in the two strains, bordered on one side by *clpB* and on the other by the type III effector *hopR* (Figures 1, 2). Broad comparisons between *Pto*DC3000 and *Pma*ES4326 further suggest that these effectors form a genomic island along with a third effector (*hopR*) across these two strains (Figure 2), an island which has been referred to as effector cluster IV in in the *Pto*DC3000 genome (Kvitko et al., 2009). Moreover, the size of the region between *clpB* and *hopR* in *Pto*DC3000 is roughly 70 kb, which closely approximates the size of each ICE in *Pma*ES4326. While this region within PtoDC3000 does contain hints that it previously housed a functional ICE, there are other sections that are quite divergent from that in *Pma*ES4326 (Figure 2). Specifically, the predicted recombinase in *Pto*DC3000 is truncated due to a frameshift mutation and multiple other loci that encode functions important for ICE conjugation and transfer are disrupted by IS elements (Figure 2). Lastly, we note that conservation of nucleotide and protein sequences between PmaICEs coupled with nucleotide diversity in *Pto*DC3000 at this genomic location renders accurate evaluation and comparison of evolutionary histories between *Pto*DC3000 and the ICEs of *Pma*ES4326 challenging. However, the most parsimonious explanation does appear that an ICE element housed both *hopQ* and *hopD* within an ancestor of strain *Pto*DC3000 and that this ICE was locked in place through both IS element insertions and loss of recombinase activity.

### An Evictable Integrative Conjugative Element Containing HopQ and HopD in *Pma*ES4326

**FIGURE 3:**
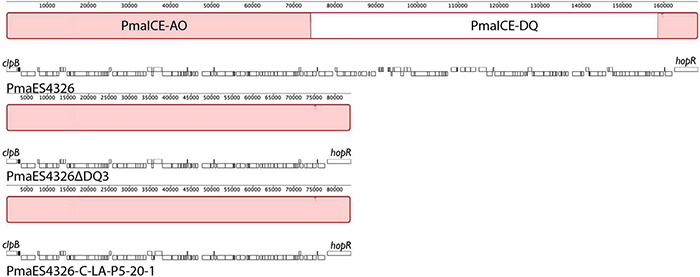
Identical deletions of PmaICE-DQ following laboratory selection and passage under selective conditions *in planta*. We compared genomic regions bordering ICE-DQ in a strain where the region containing *hopQ* was intentionally deleted (*Pma* DQ3) as well as a strain that arose after five passages *in planta* in *N. benthamiana* (*Pma* LAP5-20). Genomic segments from each of the three strains are arranged vertically, with each line labeled by strain name and genes within each strain outlined in white boxes underneath the alignment. Each contiguous segment is oriented so that *clpB* is found on the left side of the segment and so that *hopR* is found on the right side. Sections within the alignment colored red are those that are aligned into contiguous blocks by Mauve and are shared by all three strains. A white section within the wild type *Pma*ES4326 alignment represents a region (PmaICE-DQ) that is not present in the other two strains. Mauve alignments of these regions demonstrate that identical evictions of ICE-DQ occurred in each strain.

ICEs are categorized by their ability to cleanly excise from the genome, and we confirmed the prediction that the type three effectors *hopQ* and *hopD* are contained in an excisable region in *Pma*ES4326 in two distinct ways. As a first step, we characterized the size of the region excised during intentional creation of a *hopQ*- mutant. To do this, we generated a merodiploid strain in which plasmid pCPP5729 was recombined into a region adjacent to *hopQ*, and then selected for resolution of the merodiploid through plating on sucrose. Presence of *sacB* in plasmid pCPP5729 renders the merodiploid sensitive to killing by sucrose. Notably, this merodiploid also contains regions sequence identical to those surrounding *hopQ* in *Pma*ES4326 and so we originally expected that the *hopQ* gene could be locally deleted through RecA-dependent homologous recombination. We isolated sucrose resistant isolates after plating this merodiploid, and identified strains that were neither WT reverants nor clean *hopQ* deletions by PCR genotyping using previously validated genotyping primers. Interestingly, while no clean *hopQ* deletion mutants were identified after sucrose counter-selection of merodiploid strains, WT revertants were. We then performed whole genome sequencing to confirm whether the ICE-DQ genomic region was lost under these selective conditions. We refer to this strain recovered by *in vitro* selection hereafter as *Pma* DQ3. As one can see in Figure 3, *hopQ* and the surrounding regions that are predicted to be part of ICE-DQ have been lost in the targeted deletion strain *Pma* DQ3, confirming that this region can be cleanly and completely excised from the chromosome in a manner consistent with the action of site-specific recombinases contained by ICEs. Searches of both the raw Illumina and Nanopore reads for remnants of *hopQ* or *hopD* yielded no hits despite extensive depth (∼51× for Illumina reads and ∼27× for Nanopore reads), which suggests that ICE-DQ was fully lost and not retained as an episome in *Pma* DQ3 (data not shown).

### Loss of PmaICE-DQ Enables Virulence of *Pma*ES436 on *Nicotiana benthamiana*

**FIGURE 4:**
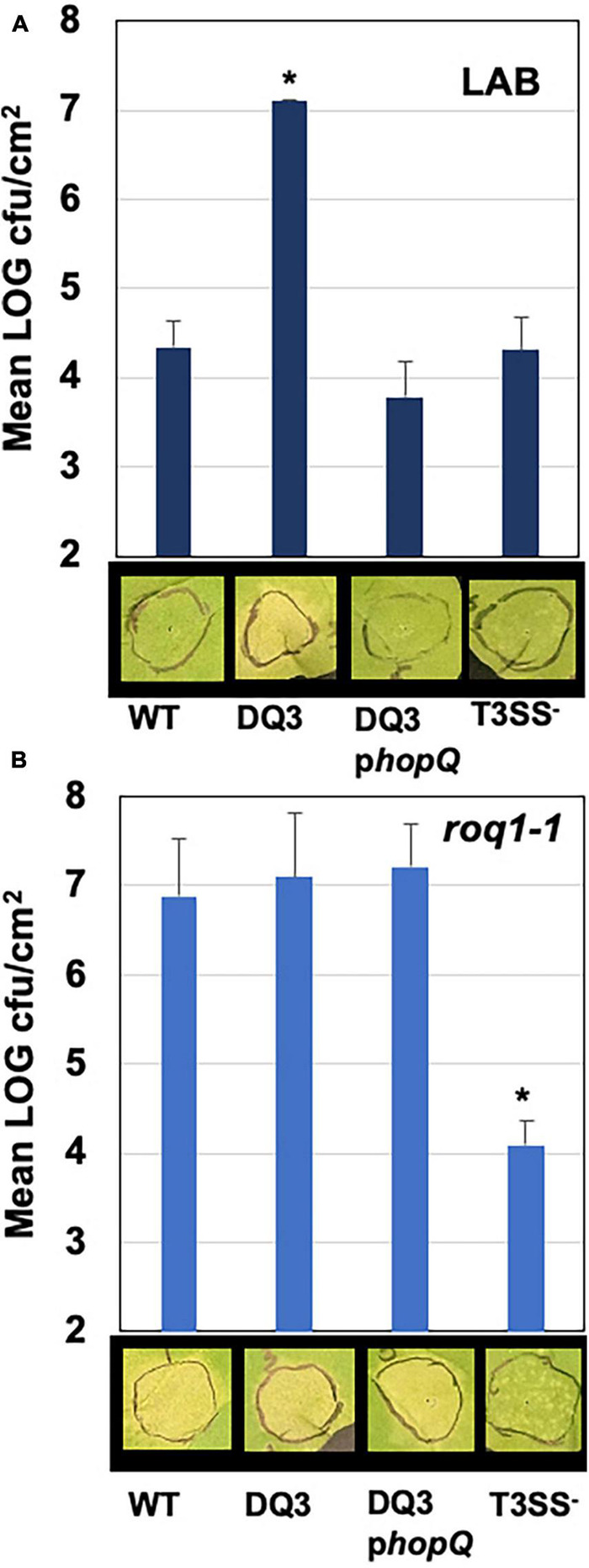
PmaES4326 gains disease compatibility with *N. benthamiana* in the absence of *hopQ*/*Roq1*-mediated ETI. The *Pma*ES4326 WT strain, *Pma* DQ3 (ΔICE-DQ), DQ3 complemented with pCPP5372hopQ (DQ3pQ), and the non-pathogenic T3SS- strain *hrcN*:Tn*5* were infiltrated into leaves of the *N. benthamiana* Lab accession **(A)** or the *roq1-1* CRISPR mutant derivative line **(B)** at 3 × 10^4^ CFU/mL. Eight days post inoculation leaves were photographed to document symptoms and bacterial load from four 4 mm diameter leaf discs from each infiltrated area by dilution plating was determined as CFUs/cm^2^ leaf tissue. Values are the means and standard deviations of LOG transformed CFUs/cm^2^ from three inoculated plants. *p < 0.05 compared to *Pma*ES4326 WT strain as determined by unpaired one-tailed *t*-test.

The presence of *hopQ* in *Pma* would be expected to elicit the ETI response in *Nicotiana benthamiana* accessions containing the R-gene *Roq1*, and a lack of compatible infection and disease. Given this information, we tested whether *Pma* DQ3 would gain compatibility with *N. benthamiana*. The *Pma* DQ3 strain did in fact gain disease compatibility with *N. benthamiana* in a manner similar to the gain-of-compatibility phenotypes previously observed for *Pto*DC3000 Δ*hopQ1-1* mutants and *Xanthomonas euvesicatoria* 85–10 Δ*xopQ* mutants (Wei et al., 2007; Adlung et al., 2016). Furthermore, *N. benthamiana* incompatibility could be restored in *Pma*ICEΔDQ3 by *hopQ* complementation *in trans* and *Pma*ES4326 incompatibility was not observed in *roq1-1* CRISPR-edited *N. benthamiana* as shown in Figure 4 (Qi et al., 2018).

### Loss of PmaICE-DQ Under Selection *in planta*

**FIGURE 5:**
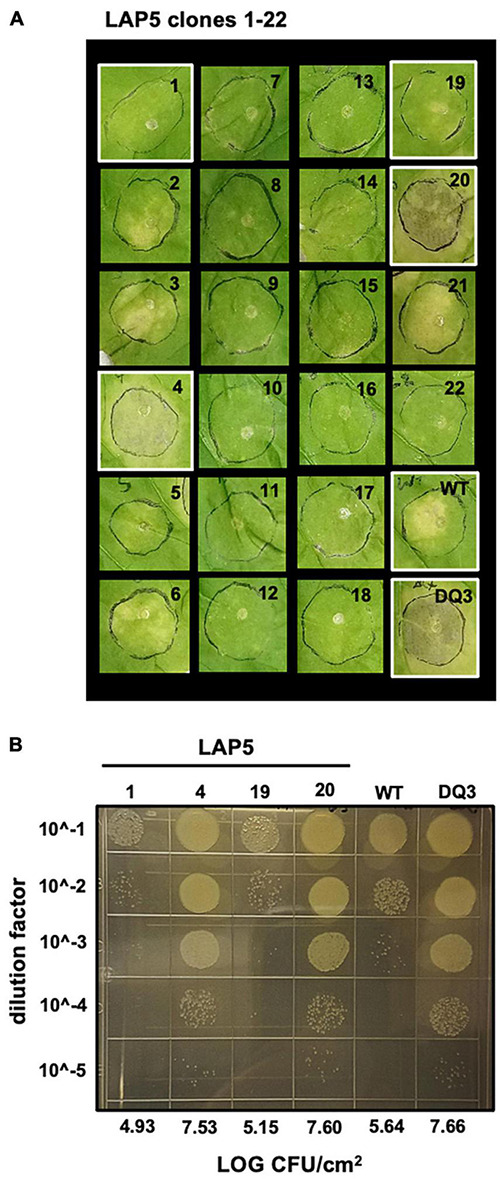
A subset of *Pma*ES4326 isolates passaged through *N. benthamiana* gain disease compatibility. **(A)** Two isolates out of 22 *Pma*ES4326 lineage A (LA) passage 5 (P5) isolates produce disease symptoms in the *N. benthamiana* LAB accession comparable to the targeted *Pma* DQ3 (ΔICE-DQ) strain. Leaves were photographed eight days post inoculation with 3 × 10^4^ CFU/mL bacterial suspensions. **(B)** Tenfold dilution series 10 μL spot plate for assessing bacterial load of select strains highlighted in white in panel A.

Observing that we could readily recover strains containing spontaneous evictions of the ICE-DQ containing a single crossover pK18 merodiploid under the selective pressure of sucrose counter-selection, and that the targeted *Pma* DQ3 strain gained disease compatibility with *N. benthamiana*, we were curious whether the selective pressure of ETI would also result in recovery of strains with ICE-DQ evicted. We inoculated *N. benthamiana* with *Pma*ES4236-C and *Pto*DC3000 at approximately 5 × 10^5^ CFU/mL establishing three lineages for each strain (Lineage A, LA; Lineage B, LB; Lineage C, LC). Six to seven days post inoculation bacteria from each lineage were recovered and used to create new inoculum to passage the bacteria through *N. benthamiana* a total of five times. Twenty-two isolated clones of each passage 5 (P5) lineage were screened for altered *N. benthamiana* disease compatibility phenotypes. For *Pto*DC3000, none of the 66 P5 clones tested produced disease symptoms consistent with the *Pto*DC3000Δ*hopQ N. benthamiana* compatible strain and thus were not examined further. However, for *Pma*ES4326, 7 of 66 (2 Lineage A, 3 Lineage B, 2 Lineage C) P5 clones were able to cause disease symptoms similar to the targeted deletion strain *Pma* DQ3 (see Figure 5A). For a subset of clones bacterial populations were determined to correlate qualitative differences in symptoms with differences in bacterial load (see Figure 5B). Genome sequencing of *Pma*ES4326 clone (*Pma*ES43226 LAP5-20) confirmed a loss of ICE-DQ in this strain identical to that observed in the *Pma* DQ3 targeted deletion strain recovered after sucrose counter-selection (Table 3 and Figure 3). We have further queried for additional evolutionary changes in *Pma*ES4326 LAP5-20 by using breseq (Deatherage and Barrick, 2014) to map Illumina reads to the *Pma*ES4326-C genome sequence. While it is possible, but not entirely clear, that a handful of additional changes have occurred in intergenic regions of this genome, there is no compelling data that any changes occurred in known virulence genes (see breseq output file on Figshare doi: 10.6084/m9.figshare.17064080).

### Transfer of PmaICE-DQ Between Strains

**FIGURE 6:**
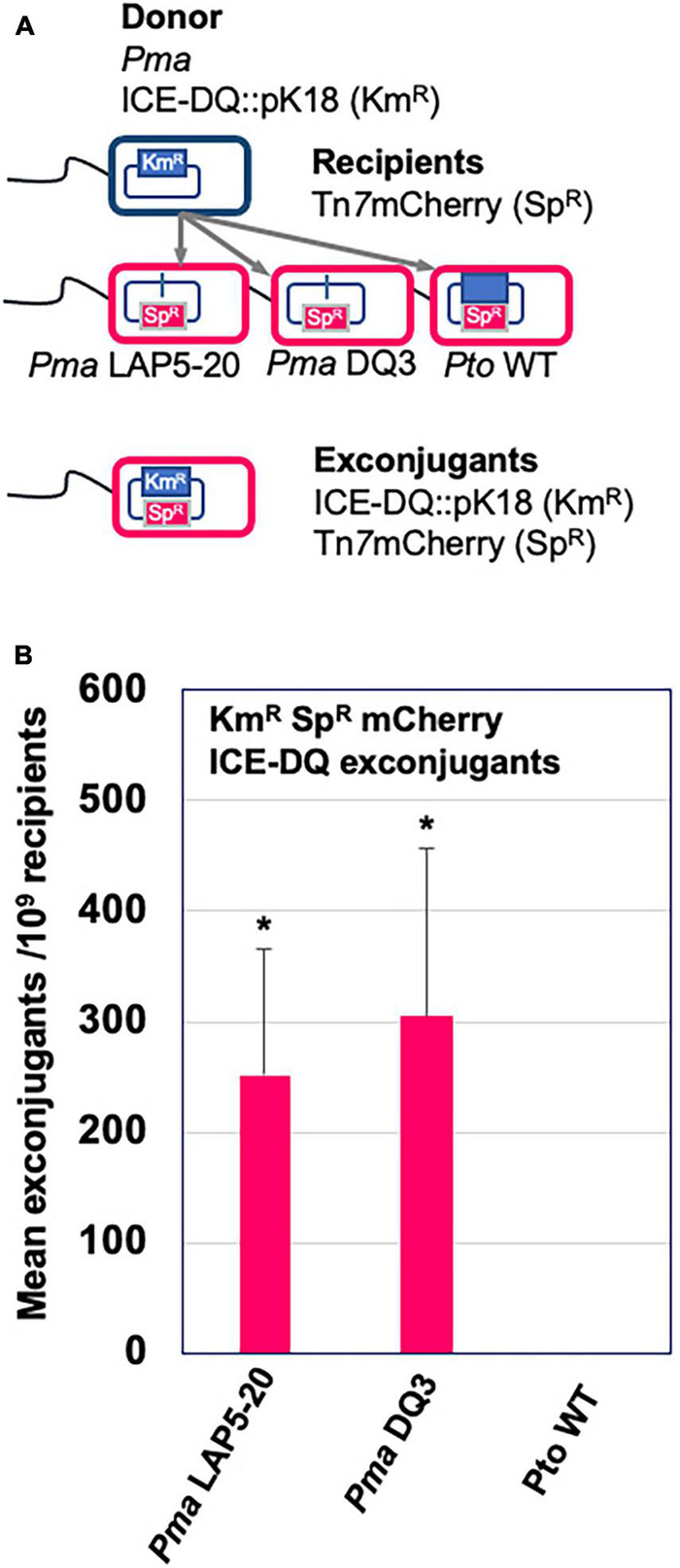
ICE-DQ can be readily transferred into *Pma*ES4326 strains that lack the ICE element. **(A)** Schematic representation of the conjugation strategy for monitoring ICE-DQ transfer between strains. The ICE-DQ of the donor strain was marked with kanamycin resistance by single crossover integration the pK18ms plasmid (*Pma* ICE-DQ:pK18). The recipient strains *Pma* DQ3 (ΔICE-DQ targeted), *Pma* LAP5-20 (ΔICE-DQ passage-derived) and *Pto*DC3000 were marked by introduction of a spectinomycin resistance Tn*7*3xmCherry transposon. Donor and recipients were co-cultured on LM media and ICE-DQ:pK18 mCherry expressing ICE-DQ exconjugants were recovered on kanamycin and spectinomycin. **(B)** ICE-DQ:pK18 exconjugant recovery frequencies/10^9^ recipients were determined based on kanamycin and spectinomycin exconjugants per spectinomycin resistant recipients as determined by dilution plating. Values are means and standard deviations of three biological replicates. *p < 0.05 compared to Pto WT strain as determined by unpaired one-tailed *t*-test.

Aside from being able to excise from the genome, ICE elements are also categorized by their ability to transfer across bacterial strains, and we therefore tested whether ICE-DQ could undergo conjugation to a naive strain. To do this, ICE-DQ was marked with kanamycin resistance by generating a single-crossover merodiploid with a pK18ms-derivative plasmid (*Pma* ICE-DQ:pK18). Three recipient strains, *Pto*DC3000 and two independent PmaΔICE-DQ strains (the targeted *Pma* DQ3 strain and the passage derived *Pma* LAP5-20) were labeled with a Tn*7* 3xmCherry resistant transposon to confer spectinomycin resistance as well as visually differentiate them from the donor strain. The *Pma* ICE-DQ:pK18, Km^R^ marked donor strain was co-cultured with each of the three Tn*7* 3xmCherry Sp^R^ recipients. Conjugations between these strains were then plated on kanamycin and spectinomycin selective media (Figure 6A). No kanamycin and spectinomycin resistant mutants or exconjugants were recovered from the *Pto*DC3000 conjugation or from the single parent control experiments. However, we were able to recover kanamycin and spectinomycin resistant mCherry + exconjugants in *Pma*ES4326ΔICE-DQ strains at rates of 2.50 × 10^–7^ stdev ± 1.23^–7^ and 3.05 × 10^–7^ stdev ± 1.51^–7^ exconjugants per recipient respectively in *Pma* DQ3and *Pma* LAP5-20 recipient strains (Figure 6B) which strongly suggests that the ICE-DQ element is readily transmissible into *Pma*ES4326 strains lacking ICE-DQ.

## Discussion

Integrative Conjugative Elements (ICEs) are important drivers of evolutionary dynamics within and across bacterial populations and communities because of their potential to disseminate genes and pathways through horizontal transfer (Johnson and Grossman, 2015). Genes encoded by ICEs often impart phenotypes critical for survival under specific environmental conditions, including antibiotic resistance loci as well as phage defense systems (Botelho and Schulenburg, 2021; LeGault et al., 2021). In the phytopathogen *Pseudomonas syringae*, plasticity of ICE elements encoding genes involved in heavy metal resistance, type III effectors, and carbon metabolism has been identified as the main difference between epidemic strains causing disease across kiwi orchards in New Zealand and with previous outbreaks (Colombi et al., 2017; McCann et al., 2017; Poulter et al., 2018). Despite the accumulation of numerous complete genomes sequences for *P. syringae* and related species and the likelihood of ICEs to impart traits that affect growth in agricultural settings, to date there have been relatively few ICEs identified and vetted for this pathogen.

Placement of genes and pathways in ICEs may be especially important under conditions of fluctuating selection pressures where selection pressures on cargo genes in an ICE switch from positive to negative depending on the environment. One such scenario involves the presence of type three effector genes, which are bacterial proteins delivered from symbionts into eukaryotic host cells and which are critical for pathogenesis of *P. syringae* strains *in planta* (Grant et al., 2006). Type three effectors are particularly well studied in phytopathogens such as *P. syringae*, where they can be of exceptional benefit on some host plants by enabling strains to overcome host immune responses but may also directly trigger immune responses based on the presence of cognate immune receptors on other host genotypes (Dillon et al., 2019a; Laflamme et al., 2020). The presence of type three effectors on ICEs would enable the rapid transmission of these critical virulence genes across strains, while also enabling genes to be rapidly lost from lineages if host genotypes shift such that the effectors are recognized by R gene pathways. Extending this thought, effectors in ICEs that are recognized by hosts are potentially lost more readily through ICE element excision than through mutation (given dedicated excision machinery of the ICEs), which may facilitate adaptation if a strain encounters a resistant host background. While not an extensive experiment, our passage experiment where a subset of *Pma*ES4326 but not *Pto*DC3000 clones becomes compatible with *N. benthamiana* does demonstrate that parameter space exists for such a scenario. Moreover, effectors that are inactivated through mutation can only be reactivated by reversion mutations or through horizontal transfer and acquisition from different strains. In contrast, if effectors are found on ICEs and lost through excision, it’s possible that a small resident pool of strains containing these ICEs will remain on other host plants or throughout the environment, facilitating rapid reacquisition of effectors that could be beneficial under different contexts. Lastly, there are a plethora of type III effectors in *P. syringae* that carry out a variety of functions across different host plants and resistance backgrounds (Dillon et al., 2019a). Some of these effectors can be considered “core” and found in syntenic locations across strains while others are more variably present. It’s possible that presence of effectors in ICEs could itself reflect something about the characteristics of these effectors, in that the proteins found as cargo on ICEs may be subject to higher levels of fluctuating selection across hosts than those found in the core set.

HopQ and HopD are often found together on genomic islands in *P. syringae* genomes, and have been associated with adaptation to agriculturally important crop plants (Wei et al., 2007; Baltrus et al., 2011; Monteil et al., 2016). Presence of *hopQ* in an active ICE in strain *Pma*ES4326 sets up an interesting scenario because a nearly identical version of this effector is present in a somewhat syntenic position in a relatively closely related strain *Pto*DC3000 except that this effector is not part of a functional ICE. Therefore, this genomic context suggests that *hopQ* would experience more evolutionary flexibility in *Pma*ES4326 than in *Pto*DC3000 because it can be more easily lost and/or transferred from this strain due to its presence in a region of the chromosome that can excised at a relatively high rate. To test for differences in evolutionary flexibility, we passaged replicate populations of both *Pma*ES4326 and *Pto*DC3000 in an accession of *N. benthamiana* which can recognize and mount an immune response to HopQ. As such, wild type versions of *Pma*ES4326 and *Pto*DC3000 are recognized by this accession and trigger an immune response which prevents disease. However, we found that passage of *Pma*ES4326 from plant to plant resulted in recovery of strains of *Pma*ES4326 that didn’t trigger the immune response. Therefore, presence of this type III effector inside of an ICE enables rapid loss of the recognized effector under conditions of negative selection and there is relatively less flexibility for this effector to be lost in a strain where it is not present in an active ICE.

One other curious feature of the genome of *Pma*ES4326 is that it encodes two distinct but highly similar ICE elements (Figure 1). The configuration is particularly interesting as two distinct ICE elements have integrated successively into the *Pma*ES4326 genome and many of the genes involved in ICE functions are quite conserved between the two. It also appears as though integration of the first element recreated a tRNA with a proline anticodon and that the second ICE element can use this as a further integration point. Indeed, excision of ICE-DQ in both the targeted *Pma* DQ3 and passage-derived *Pma* LAP5-20 strains reported here also deletes one of the predicted proline tRNAs. It may therefore be no coincidence that two ICE elements that are highly similar (but which contain different cargo regions) can integrate successively into the genome as it’s straightforward to imagine that highly specific recombinases would have similar target sequences.

Although not the main focus of this manuscript, we also report on plasticity of the *Pma*ES4326 genome writ large across lab derived strains (Table 2). As the number of complete *P. syringae* genome sequences accumulates, it is becoming more apparent that strain *Pma*ES4326 is notable compared to the rest of the species complex because it contains so many secondary replicons as well as additional elements that contribute to genomic plasticity (Stavrinides and Guttman, 2004; Stavrinides et al., 2012). Many of these plasmids appear to contain genes involved in virulence and so acquisition of these replicons through horizontal gene transfer has likely contributed to the pathogenic ability of this strain across multiple hosts (Stavrinides and Guttman, 2004). However, there is a clear downside to the large plasmid repertoire of *Pma*ES4326 as demonstrated by genome sequences reported here. Although we can’t definitively catalog passage histories, genome sequences from isolates from the Dangl and Collmer labs are likely not diverged by > 10 passages total. In this time, the Dangl isolate (*Pma*ES4326-D) has lost two different plasmids that were originally reported by Stavrinides et al. and which are contained in the Collmer lab isolate (*Pma*ES4326-C). Indeed, genome assemblies for the targeted ICE-DQ excision strain *Pma* DQ3 suggest that this strain has also lost at least one plasmid in addition to the ICE-DQ after only three additional passages in culture. Lastly, even though this strain was extensively surveyed for plasmids through gel electrophoresis and Sanger sequencing, we report the existence of an additional large plasmid present within all sequenced isolates of this strain.

## Data Availability Statement

The datasets presented in this study can be found in online repositories. The names of the repository/repositories and accession number(s) can be found in the article/supplementary material.

## Author Contributions

DB and BK conceived, conceptualized, analyzed the experiments, and co-wrote the manuscript. QF conducted a subset of experiments described in the manuscript. All authors contributed to the article and approved the submitted version.

## Conflict of Interest

The authors declare that the research was conducted in the absence of any commercial or financial relationships that could be construed as a potential conflict of interest.

## Publisher’s Note

All claims expressed in this article are solely those of the authors and do not necessarily represent those of their affiliated organizations, or those of the publisher, the editors and the reviewers. Any product that may be evaluated in this article, or claim that may be made by its manufacturer, is not guaranteed or endorsed by the publisher.

## Footnotes

1
http://n2t.net/addgene:48831
